# Desmoplastic Small Round Cell Tumor of the Abdomen and Pelvis: Clinicopathological Characters of 12 Cases

**DOI:** 10.1155/2014/549612

**Published:** 2014-06-02

**Authors:** Guangzhao Zhang, Guangjun Liu, Dahua Zhao, Xijun Cui, Gang Li

**Affiliations:** ^1^Department of General Surgery, Wuqing District People's Hospital, Tianjin 301700, China; ^2^Department of General Surgery, Wendeng Central Hospital, Shandong, China; ^3^Department of Pathology, Affiliated Hospital of Binzhou Medical University, Binzhou, Shandong, China; ^4^Department of Urology, Second Hospital of Tianjin Medical University, Tianjin Institute of Urology, Tianjin 300211, China

## Abstract

*Purpose*. To study the clinical, radiological, and pathological characteristics of abdominal desmoplastic small round cell tumor (DSRCT) and investigate the optimal therapy modalities. *Patients and Methods*. A retrospective cohort study was performed on 12 abdominal DSRCT patients; all pathological, radiological, and prognostic data were analyzed. There were 3 patients (25%) with metastatic disease at presentation. In all 12 cases, 6 cases underwent operation and adjuvant chemotherapy (group 1, 6/12, 50%). The other 6 cases were diagnosed by fine needle aspiration or exploratory laparotomy biopsy (group 2, 6/12, 50%); all cases received four to six courses of multiple agents chemotherapy, respectively. *Results*. All cases were finally diagnosed as DSRCT pathologically. Among group 1, all cases underwent en bloc resection (2/6, 33%) or tumor debulking (4/6, 67%) and, following four courses of multiple agents chemotherapy, Kaplan-Meier analysis revealed that 3-year survival was 50% in group 1 versus 16.7% in group 2 (*P* < 0.05). Gross tumor resection was highly significant in prolonging overall survival; patients with localized solitary lesion have a better prognosis, most likely due to increased feasibility of resection. *Conclusions*. DSRCT is a rare malignant tumor with poor prognosis. Surgical excision with combination chemotherapy as an adjunct is mandatory for nonmetastatic cases because these modalities used in isolation may have less impact.

## 1. Introduction

Desmoplastic small round cell tumor (DSRCT) is an extremely rare, highly aggressive, and malignant neoplasm initially reported by Gerald and Rosai in 1989 [1]. The pathogenesis or histogenesis of DSRCT is uncertain; it mainly occurs in adolescents and mostly involves the abdominal and/or pelvic peritoneum [2]. Moreover, it was also reported in epididymis, pleura, soft tissues, bone, ovarian, and kidney [3–10]. The diagnosis can be confirmed by histological and immunohistochemistry studies. Despite multimodality treatments, optimal treatment strategies remain controversial and the prognosis is poor. Current multimodality treatment rarely achieves cure and prolongs life. Here, we described 12 cases of abdominal DSRCT and retrospectively analyzed its clinical, radiological, and biopathological features, highlighting the modalities of treatment.

## 2. Subjects and Methods

Institutional review board approval was obtained for this retrospective cohort study. It was performed on 12 cases of DSRCT between March 2003 and May 2011. The clinical, pathological, and radiological imaging studies were available for review. In our series, the median age was 26.4 ± 8.4 years with a range of 14–39 years and the male-to-female ratio was 2 : 1. All 12 patients were evaluable for radiological characteristics and histological and immunochemical data. All cases underwent abdominal and pelvic CT examination with contrast-enhanced CT scan. According to their treatment, two subgroups were defined: group 1 (nonoperation group, 6 cases); all were treated with multiple agents chemotherapy following four to ten courses of multiple agents chemotherapy until tumor progression; group 2 (operation group, 6 cases) underwent exploratory laparotomy, followed by en bloc resection in two cases and debulking or cytoreductive surgery in four cases, because the lesions were found as multiple nodes in different sizes and distributed in multiple organs including peritoneum, mesentery, and pelvic cavity. Debulking surgery in our investigation is defined as definitive removal of at least 90% of the tumor burden. Because of multiple lesions or diffuse nature of tumors infiltrating adjacent vital organs, it is often impossible to complete resection of all tumors with negative microscopic margins. Subsequently, all cases were treated with adjuvant chemotherapy. In both groups, the chemotherapy scheme was based on the intensive use of vincristine (1.5 mg/m^2^, day 1), ifosfamide (3 g/m^2^/day × 3), and doxorubicin (30 mg/m^2^/day × 2) or the IVC scheme (ifosfamide 3 g/m^2^ days 1 and 2, vincristine 1.5 mg/m^2^ day 1, and cisplatin 120 mg/m^2^ days 1–3) for four to ten cycles. Response to chemotherapy based on the degree of tumor volume reduction was defined as follows: complete response (CR), partial response (PR), stable disease (SD), and progressive disease (PD). Survival outcomes were estimated using the Kaplan-Meier method and compared between groups by the use of log-rank test. *P* value <0.05 was considered to indicate statistical significance; all statistical tests were carried out utilizing SPSS, version 17.

## 3. Results

In our series, the most common primary manifestations were gastrointestinal symptoms including nausea, vomiting or distention (*n* = 8), palpable abdominal mass (*n* = 6), and urinary disorders (*n* = 2) and no positive findings (*n* = 1). Serum CA125 was examined in three cases but only one increased significantly about 131 U/ML whose normal value is 1.9~16.3 U/ML. General information of all patients and follow-up data are listed in Table 1.

### 3.1. Image Findings

**Figure 1:**
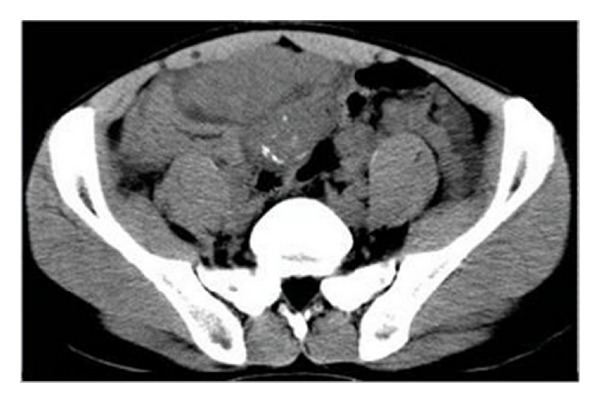
Abdominopelvic CT scan revealed diffuse multiple soft-tissue masses in peritoneal and mesenteric surfaces.

**Figure 2:**
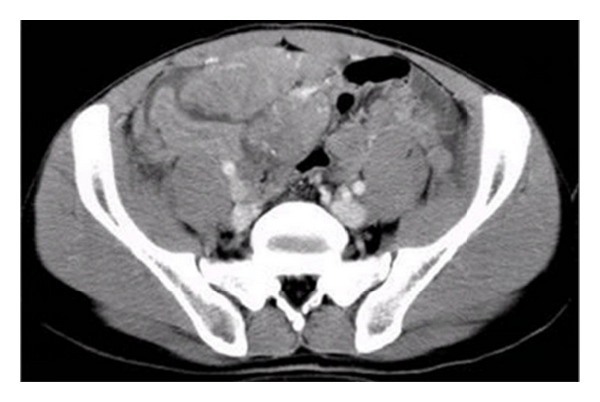
Contrast-enhanced CT scan revealed that most masses were slightly enhanced.

**Figure 3:**
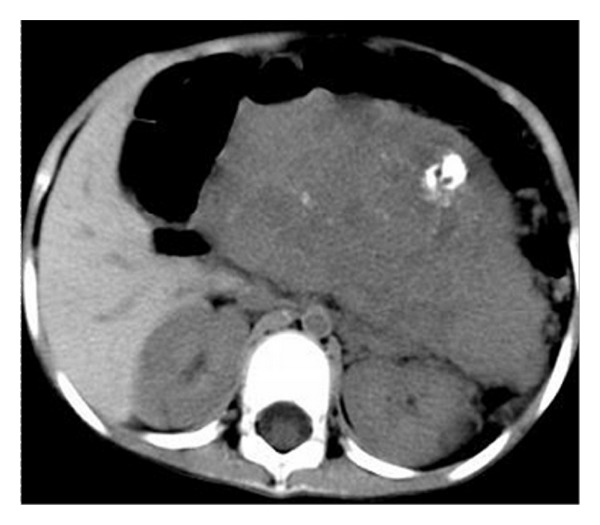
Axial unenhanced abdominopelvic CT showed a large, solid, and heterogeneous mass with scattered calcifications.

**Figure 4:**
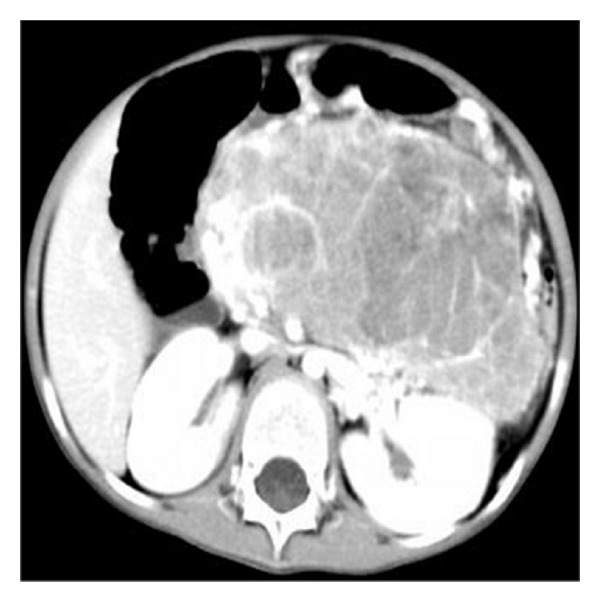
Contrast-enhanced CT revealed the heterogeneous mass with obvious enhancement areas and scattered low attenuation.

All patients underwent abdominal and pelvic CT examination: three cases complicated with hydronephrosis, two cases had uterine accessories infringement, and three cases presented with liver or lung metastases. According to striking CT features, the abdominal and pelvic lesions were divided into two groups. The most common imaging finding was multiple nodular peritoneal soft-tissue masses with variable sizes (group 1, *n* = 9, mean number, 4.7; range of 1–10 cm) in abdominal and pelvic space (Figure 1); among those, only three (33.3%) cases displayed heterogeneous enhancement after IV contrast administration (Figure 2). Isolated tumor was relatively well-defined (group 2, *n* = 3, with a mean diameter of 10.8 cm and range of 5–16 cm) in abdominal-pelvic space (Figure 3). All dominant tumors displayed heterogeneous enhancement after IV contrast administration (Figure 4). The tumors were predominantly intraperitoneal (*n* = 7), located in the omentum and paravesical region (*n* = 5). CT also showed serosal tumor implants from intraperitoneal spread (*n* = 3). Two patients had hydronephrosis (unilateral in two cases and bilateral in one); one patient has frequency and dysuria. Areas of central low attenuation within tumors were seen in 4 patients. Scattered amorphous or punctuate tumor calcification was seen in three patients (25%). On contrast enhancement CT scan, it was modest enhancement (*n* = 4), obvious enhancement (*n* = 5), without enhancement (*n* = 3).

### 3.2. Pathology Results

**Figure 5:**
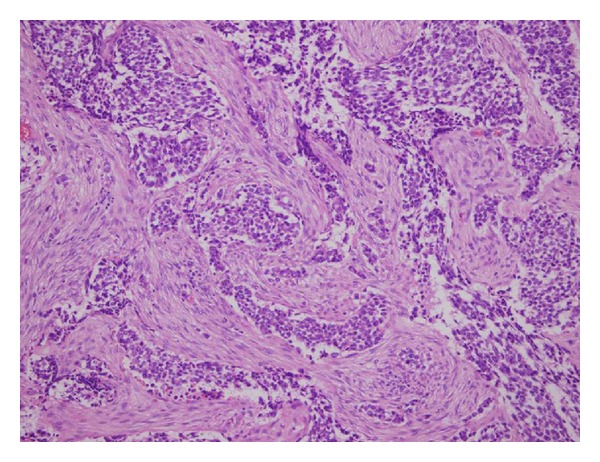
Pathology investigation showed nests of small, round undifferentiated cells separated by myxomatous desmoplastic stroma (haematoxylin-eosin stain, original magnification, ×20).

**Figure 6:**
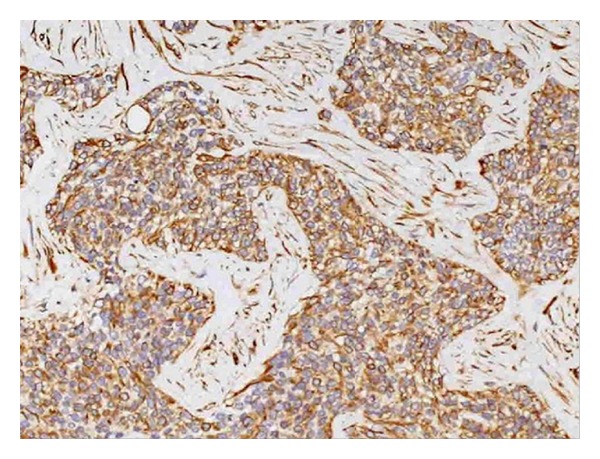
Immunohistochemical staining the cells demonstrated expression of EMA (original magnification, ×20).

Grossly, the mass showed the presence of nonuniform white-gray multinodules that were widely distributed in the peritoneum. Pathology study revealed that the tumor is characterized by sharply demarcated nests of relatively small cells embedded in a cellular desmoplastic stroma; the tumor cells were round or oval in shape with thick nuclear chromatin and few cytoplasm (Figure 5). Immunoperoxidase stain in these cases was positive for vimentin, keratin, desmin, PCK, NSE, and EMA (Figure 6) and negative for S-100, CMA, and 34*β*E.

### 3.3. Prognosis

**Figure 7:**
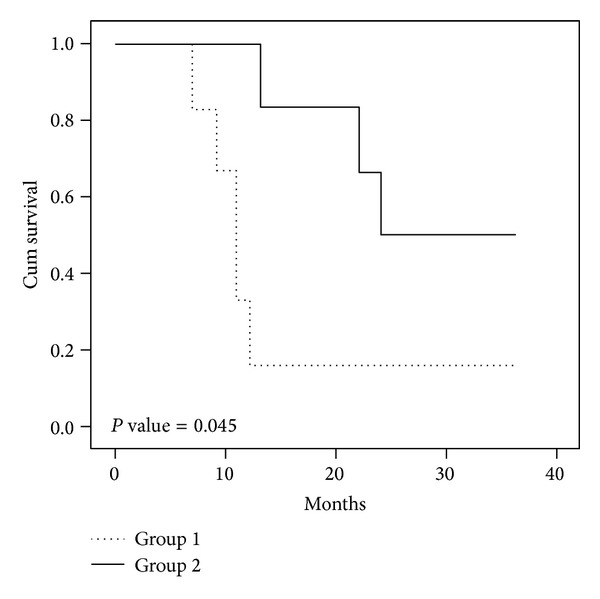
Kaplan-Meier analysis revealed that the 3-year survival was 50% in group 1 versus 16.7% in group 2.

In group 1, all six patients were evaluable for response and followup for 36 months, 1 patient had PR, 3 patients had stable disease, and 2 patients had progressive disease. Tumor regression has been noted during multiagent chemotherapy, but response is often of brief duration. All patients died of tumor progression or widespread metastases 7, 9, 11, 11, 12, and 37 months after diagnosis. In group 2, 4 patients died of tumor relapse or widespread metastases 13, 22, 24, and 40 months after diagnosis. Only two cases with complete tumor resection were alive without obvious residual tumor with a followup for 36 and 42 months after surgery. Complete tumor resection was an independent prognostic factor and significantly correlated with long survival. Kaplan-Meier analysis revealed that the 3-year survival was 50% in group 1 versus 16.7% in group 2 (*P* < 0.05) (Figure 7).

## 4. Discussion

The rarity of DSRCT may attribute to the less knowledge of its biological behavior; meanwhile, the pathogenesis of DSRCT is unclear. Histologically, the majority of DSRCTs are distinguished by solid clusters of undifferentiated small round cells embedded in dense desmoplastic stroma [11–13]. These tumors are also characterized by polyphenotypic differentiation as evidenced by immunohistochemical staining for epithelial, mesenchymal, and neural markers including cytokeratins (EMA, AE1/3, and CAM5.2), desmin, vimentin, and neuron-specific enolase (NSE) [14–16]. DSRCT belongs to the family of “small round blue cell tumors”; nevertheless, molecular biology has proved that DSRCT is a unique tumor which is different from other types of small round cell tumor. The genetic characterization of DSRCT is a chromosomal translocation of t(11;22)(p13;q12) between Ewing's sarcoma (EWS) gene on chromosome 22 and Wilm's tumor (WT1) gene on chromosome 11, leading to a EWS-WT1 fusion transcript; the characteristic translocation t(11;22)(p13;q12) is specific for DSRCT, regardless of its site [11, 16]. This fusion product causes a loss of the tumor suppressor function of WT1 and a putative upregulation of various families of growth factors from the EWS gene [17].

DSRCT mainly affects young adolescents with a male-to-female ratio of 4 : 1 and tends to be symptomatic on presentation. In our series, the average age is relatively older than the literature reported. The tumor has a predilection for the omentum and adheres to the hollow viscus, surface of the omentum, mesentery of bowel, or pelvis peritoneum. Digestive organs involvement is secondary; liver and lung are two common sites for metastatic disease beyond the peritoneum. Extra-abdominal primary DSRCTs are relatively uncommon. Clinically, patients may be asymptomatic for long periods of time and diagnosis is made when tumor burden is large. Some abdominal DSRCTs present with persistent, nonspecific abdominal symptoms such as discomfort or distention, constipation or bowel obstruction, nausea or emesis, weight loss, ascites, increasing abdominal girth, and palpable masses and infiltration of urinary organs such as hydronephrosis or urinary disorders. Occasionally, incidental palpable abdominal masses may be the first presentation. Clinical presentation may be related to tumor size, distant metastasis, and infiltration of the surrounding structures. In our series, the median tumor size was 8.4 ± 5.3 cm. Although it can develop at various sites, most cases usually present with widespread abdominal serosal involvement whose growth pattern closely mimics that of mesothelioma; it is speculated that the cell origin of DSRCT may be a primitive mesothelial cell. Typical cases of DSRCT in the intra-abdominal cavity or gastrointestinal tract are accompanied by abdominal mass and/or pain which are similar to gastrointestinal tumors. Ascites and solitary or multiple nodules can also present in some patients. Occasionally, urinary tract symptoms were caused by ureter or bladder involvement, whereas DSRCT originating from the genitourinary tract system was rare. Serum CA-125, which is reported to be increasing significantly among DSRCT patients, may be used as a marker but lack specificity [18]. Only one in our series with serum CA-125 obviously increased to 131 U/ML (normal value 1.9 U/ML to 16.3 U/ML).

CT scan is the most widely used diagnostic modality; abdominopelvic site was the commonest presentation and the disease can occur at other nonserosal surfaces also. CT frequently shows multiple bulky, lobulated, heterogeneous, and peritoneal soft-tissue masses with a predilection for intraperitoneal spread without obvious primary organ involvement. In advanced cases, abdominopelvic DSRCT can develop into bulky and multiple masses that displace the neighboring organs. These radiographic findings must be distinguished from peritoneal carcinomatosis. The hallmark imaging feature was from dozens to hundreds of multiple millimeter sized nodules to lobulated or confluent peritoneal masses which lack characteristic features, and preoperative imaging had a low diagnostic utility. Bellah et al. analyzed CT characteristics of 11 patients with DSRCT and found that most characteristic CT features of DSRCT include bulky intra-abdominal soft-tissue masses that involve omental and serosal surfaces, without a distinct organ of origin, and widespread implant of the tumor was also found [19]. In the early stage of DSRCT, the tumor appeared as single or multiple nodules. However, in our series, the most common character was found to have multiple lobulated solid nodules with irregular boundary and widely distributed on the peritoneum. The hypodense areas and heterogeneity reflect tumor hemorrhage or necrosis. Ascites, calcifications, nodular peritoneal thickening, lymphadenopathy, hydronephrosis, and bowel obstruction were associated findings. Bulky peritoneal soft-tissue masses without an apparent organ-based primary site are characteristic of intra-abdominal desmoplastic small round cell tumor [20]. The most useful radiographic method is CT scan with intravenous contrast. The typical imaging reveals multiple low-attenuation peritoneal soft tissue with regular contour. Most masses are located within mesentery, omentum, and paracolic gutter or along abdominopelvic peritoneal surfaces. Tumors without an apparent primary organ-based distribution can be suspicious for DSRCT. Although the findings are nonspecific, this diagnosis can be considered in young adults. Solid components may be mildly enhanced and related to the densely packed cells and desmoplastic stroma. On abdominal MRI, DSRCTs often appear as lesions with heterogeneous isointense or hypointense areas on T1-weighted images and heterogeneous hyperintense on T2-weighted MR images [21]. Radiologically, DSRCT is similar to other intra-abdominal tumors, especially those presenting within visceral organs, so nonspecific radiological features mandate pathological diagnosis.

Proper consensus about treatment has not yet been established, and the treatment of DSRCT remains a clinical challenge and lacks standard treatment modalities. Despite multiple treatment strategies including high-dose chemotherapy regimens active for DSRCT, aggressive debulking surgery, whole abdominal radiation, or even autologous stem cell transplant, the prognosis of DSRCT is poor and most cases die within 3 years [22].

Owing to frequent multiple peritoneal implants or multifocal lesions, complete resection without sacrificing the adjacent organs is usually impossible. The effect of complete resection of disseminated tumors on survival is still unknown because of the rarity of achieving complete resection at operation.

For advanced disease, symptom palliation is paramount as these modalities impact survival minimally. Aggressiveness of DSRCT may add a surgical burden and the impact of surgical resection upon survival remains unclear; in our series, patients who underwent complete surgical excision seem to provide a better survival. Nevertheless, larger prospective studies are needed to provide it. Cytoreductive or debulking surgery has been performed before chemotherapy is used for symptomatic relief, especially for those intestinal obstruction cases. When peritoneum was involved, the patient should be performed peritonectomy and re-operation was needed occasionally. In our experience, surgical resection especially total radical resection or even debulking combined with multiagent adjuvant chemotherapy can highly improve overall survival of advanced DSRCT. Although it is difficult to demonstrate, increased survival depends only on combined modality therapy instead of other factors.

Many chemotherapy combinations have been tried, but the optimal scheme and generally accepted chemotherapy option have not been determined at present. In previous investigation, DSRCT has been confirmed to be moderately sensitive to intensive chemotherapy; unfortunately, response duration was extremely poor [23]. Some found that rapamycin can make cancer cells stop in G1 phase. Its derivatives RAD001 and CCI-779 already have been performed in the clinical trials of phases I and II, respectively, and can be regarded as a cytotoxicity drug therapy for DSRCT [24]. Bertuzzi et al. reported that 7 patients with DSRCT received induction chemotherapy with ifosfamide, epirubicin, and vincristine. Single- and multiagent chemotherapy trials have yielded moderate results. The overall survival of DSRCT is approximately 30% to 55% despite chemotherapy, radiotherapy, and aggressive surgical resection [25, 26]. Many aggressive combination chemotherapy regimens have been trialed in DSRCT but none have shown curative outcome [27]. DSRCT is too rare to establish chemotherapy guidelines on the basis of the published medical literature and our initial experience. Moreover, randomized trials comparing high-dose chemotherapy or chemotherapy plus surgery to chemotherapy alone are impossible to carry out. More efforts to prolong survival and produce a symptomatic benefit are justified. In our initial experience, if the tumor is too extended to be radically excised, the patient should start chemotherapy. Unfortunately, the response of DSRCTs to conventional chemotherapy is poor or temporarily effective, and its impact on overall survival remains to be determined; meanwhile, the optimal chemotherapy modalities remain to be determined. The survival benefit from chemotherapy may outweigh its side effect profile.

Radiation treatment in DSRCT is controversial; radiotherapy especially whole abdominal-pelvic radiotherapy (WAPI) in DSRCT has not been used extensively owing to its acute toxicities and low response rate [28]. However, some researchers believed that WAPI has certain effect. Goodman reported that 21 patients with DSRCT underwent WAPI radiotherapy; after maximal surgical debulking, patients were treated with external beam radiotherapy to the whole abdomen and pelvis to a dose of 30 Gy. The median followup was 28 months and the overall survival rate at 3 years was 48% [29].

Initial data confirmed that the combination with continuous hyperthermic peritoneal perfusion may be a rational approach to improve local control of abdominal DSRCT [30]. Recently, the largest published series of HIPEC therapy to date proved that complete cytoreduction and HIPEC can improve survival of DSRCT [31]. The influence of other salvage therapies, such as immunotherapy or bone marrow ablation, is still undetermined. Initial investigation revealed that autologous stem cell transplantation is useful in prolonging survival, even in patients with residual or persistent disease before transplant, most cases underwent high-dose chemotherapy, and the role of autologous stem cell transplantation and high-dose chemotherapy remains unclear, so the role of autologous stem cell transplantation is not clear or determined. Recent studies revealed that some targeted therapeutic agents can be used to treat such lesions [32]; this may be a point for further prospective research and it is reasonable to prompt patient enrollment in clinical trials.

## 5. Conclusions

Management of DSRCT remains challenging and current schemes lack a significant cure rate despite the use of aggressive treatments. According to the current series, we would recommend aggressive debulking plus multiple agent chemotherapy for advanced abdominal DSRCT patients. The demerits of this investigation are the absence of randomized and large-scale trials. Our knowledge is based on a small series of patients in whom the outcomes are highly variable depending on the extent of the disease, resectability, and type of therapy.

## Figures and Tables

**Table 1:** Clinical and CT characteristics of abdominal DSRCT.

Number	Sex/age (years)	Symptoms/signs	Tumor localization	Treatment modality	Survival and outcome (months)
1	F/18	Palpable abdominal mass, distention	Mesentery pelvic cavity	Biopsy, chemotherapy	7
2	M/14	Pain, vomiting palpable mass	Omentum mesentery pelvic	Biopsy, chemotherapy	9
3	M/23	Abdominal pain, urinary retention	Colon, pelvic concurrent metastasis	Biopsy, chemotherapy	11
4	M/18	Vomiting, palpable mass	Mesentery, abdomen, involving liver.	Biopsy, chemotherapy	11
5	M/38	Abdominal pain distention	Mesentery, intestines	Biopsy, chemotherapy	12
6	M/54	Vomiting	Intestines, mesentery	Unresectable, chemotherapy	37
7	F/18	Abdominal pain, palpable mass	Retroperitoneum, abdomen	Multiple-organ en bloc resection chemotherapy	13
8	F/16	Vomiting	Omental and serosal surfaces	Surgery complete chemotherapy	22
9	M/24	Nausea, palpable mass	Intestines, pelvis, and retroperitoneum	Debulking surgery, chemotherapy	24
10	F/26	Abdominal distention	Abdomen, retroperitoneum	Surgery (microresiduals), chemotherapy	36
11	M/21	Abdominal pain, hydronephrosis	Widespread abdomen, peritoneum	Cytoreductive surgery, chemotherapy	40
12	M/39	No symptom	Omentum	Complete resection, chemotherapy	Followup 36, no evidence of disease

F: female, M: male.
